# Native T1 mapping versus CMR Feature Tracking (FT) derived strain analysis for the assessment of cardiac disease manifestation in Anderson Fabry

**DOI:** 10.1186/1532-429X-18-S1-Q43

**Published:** 2016-01-27

**Authors:** Anna B Reid, Christopher A Miller, Ana Jovanovic, Peter Woolfson, Nik Abidin, Richard P Steeds, James Moon, Matthias Schmitt

**Affiliations:** 1 grid.5465.20000000404309363Cardiology, University Hospital of South Manchester, Liverpool, United Kingdom; 2 grid.412346.60000000102372025Metabolic Medicine, Salford Royal Foundation Trust, Manchester, United Kingdom; 3 grid.412346.60000000102372025Cardiology, Salford Royal Foundation Trust, Manchester, United Kingdom; 4 grid.412563.70000000403766589Cardiology, University Hospitals Birmingham, Birmingham, United Kingdom; 5 grid.439749.4Cardiology, University College London Hospitals, London, United Kingdom

## Background

Cardiovascular sequelae represent a leading cause of mortality in Fabry disease. Early detection of cardiac involvement is therefore a subject of considerable interest. Low native T1 values have been shown to be associated with echocardiographic markers of early systolic and diastolic dysfunction, even in those without left ventricular hypertrophy (LVH), and conceptually could be used to optimise the efficacy of Enzyme replacement therapy (ERT). The purpose of this study was to determine the relationship of both, native T1 and feature tracking CMR (FT-CMR) derived strain parameters, with maximal LV wall thickness and LVmass in Fabry's as a clinical marker of cardiac disease manifestation.

## Methods

66 genetically confirmed successive Fabry patients (24 of which ERT naïve, 3 male, 21 female) underwent CMR at 1.5T (Avanto, Siemens AG, Erlangen, Germany). A standardised clinical CMR protocol including T1 mapping (Siemens Molli WIP 448) was used in all. A single ROI was selected in the mid-wall of a basal and mid-short axis slice, to determine a native T1 value, which was then corrected for heart rate, and averaged. Global longitudinal (GLS) and circumferential strain (GCS) parameters were derived from 4-chamber and corresponding mid-short axis SSFP cine images, using dedicated CMR feature tracking software (Diogenes® TomTec, Germany). Maximal wall thickness was determined from the mid short axis slice, and LV mass was calculated from the short axis stack (CMR Tools). Patients were subdivided into two groups of normal LV mass and increased LV mass.

## Results

**Figure 1:**
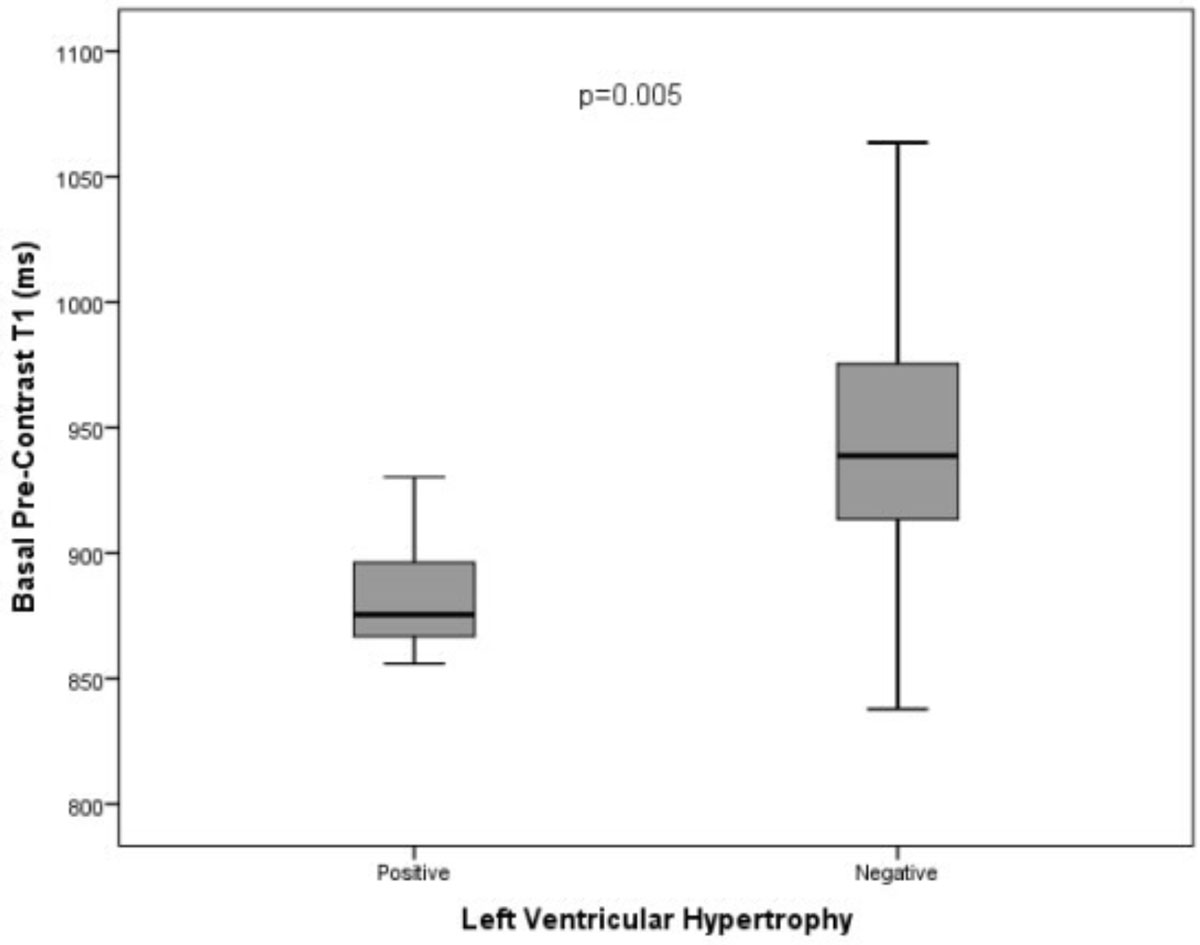
**Basal pre-contrast T1 values in LVH positive and negative patients**.

Baseline characteristics are shown in table 1. Basal T1 values were significantly lower in those with increased LV mass compared to those with normal LV mass (901 ± 36 ms vs. 951 ± 59 ms, p = 0.002, figure 1). GLS was also seen to be reduced in this group (-14.1 ± 6.7 vs. -20.3 ± 3.7, p = 0.002). In those with normal LV mass, T1 values were lower in males (924 ± 59 ms vs. 964 ± 55 ms, p = 0.03) whilst there was no significant difference in GLS (-11.8 ± 5.7 vs. -16.4 ± 7.3, p = 0.669).

**Table 1:** Baseline Characteristics

	Male (n = 24)	Female (n = 42)	
Age (years)	44.5 ± 13.5	45.9 ± 14.3	0.686
LV Mass BSA (g/m2)	98.8 ± 53.7	57.1 ± 18.3	0.001
Ejection Fraction (%)	66 ± 7	66 ± 8	0.794
Maximal Wall thickness (mm)	14.5 ± 6.8	9.6 ± 2.5	0.002
Systolic blood pressure (mmHg)	125.1 ± 12.5	119.7 ± 20.8	0.264
Diastolic blood pressure (mmHg)	72.5 ± 9.6	70.1 ± 12.7	0.436
Pre-contrast basal septal T1 (ms)	912 ± 53	954 ± 56	0.004
Pre-contrast mid septal T1 (ms)	915 ± 48	952 ± 52	0.007
Pre-contrast mean septal T1 (ms)	914 ± 48	953 ± 51	0.003
Global Longitudinal Strain (%)	-16.3 ± 5.7	-20.2 ± 5.0	0.007
Global Circumferential Strain (%)	-25.0 ± 4.9	-24.0 ± 5.2	0.49

There was moderate negative correlation between LV mass and basal T1 values (rs = -0.500, p < 0.0001), with only mild positive correlation between mass and GLS (rs = 0.348, p = 0.004). Native T1 values did not correlate with GLS (basal pre-contrast T1: rs = -0.203, p = 0.103). In those with normal LV mass, basal T1 values correlated better with LV mass as compared to GLS (: rs = -0.365, p = 0.009 and rs = -0.012, p = 0.932, respectively), whereas the opposite was seen in those with increased LV mass.

## Conclusions

Native T1 mapping shows stronger correlation with increased LV mass as compared to FT-MRI derived longitudinal strain. It shows promise as a potential future biomarker to detect early cardiac involvement in Fabry disease, and potentially to guide patient selection and timing of therapy.

